# Euthymic patients with predominantly manic polarity avoid happy faces in a dot probe task

**DOI:** 10.1186/s40345-022-00262-8

**Published:** 2022-06-23

**Authors:** Martina Wenzel, Heike Althen, Julia Veeh, Andreas Reif

**Affiliations:** 1 Department of Psychiatry, Psychosomatic Medicine and Psychotherapy, University Hospital, Goethe University, Frankfurt, Germany; 2 Department of Psychiatry, Psychotherapy and Psychosomatic Medicine, University Hospital, University of Würzburg, Würzburg, Germany; 3 Department of Neurology, Technical University of Munich (TU), Munich, Germany

**Keywords:** Bipolar disorder, Dot-probe task, Attentional bias, Predominant polarity, Emotion regulation, Euthymic phase

## Abstract

**Background:**

Some studies suggest a mood-congruent attentional bias in bipolar patients. However, for euthymic patients, especially in dependence on the predominant polarity, there is little and inconsistent data. A clearer understanding of emotion-related attentional biases and their relationship to dysfunctional emotion regulation could help improving the diagnostics and treatment of bipolar disorder (BD). Twenty bipolar patients in a depressive state (BP-acute-D), 32 euthymic patients with manic (BP-euth-M) or depressive (BP-euth-D) predominant polarity, and 20 healthy control participants (HC) performed a dot-probe task (DPT) with happy and sad faces presented for 250 ms or 1250 ms in two different runs. Emotion regulation strategies were assessed with two questionnaires.

**Results:**

In the short presentation condition of the DPT, BP-euth-M showed less attention for happy faces than HC (p = .03, r = − 0.48). BP-acute-D scored lower in cognitive reappraisal and putting into perspective and higher in suppression, catastrophizing, and rumination than HC. BP-euth-M scored higher in rumination and BP-euth-D lower in putting into perspective and higher in catastrophizing than HC. In BP-euth-D and HC, bias scores for sad faces in the longer presentation condition and reappraisal scores correlated positively.

**Conclusions:**

Results of the DPT suggest an avoidance of happy faces for BP-euth-M which we interpret as a protection mechanism for triggers of mania. That individuals who apply more reappraisal show more selective attention to sad faces could on the one hand reflect a mental effort in reevaluating the sad emotional input and on the other hand a greater tolerance for it.

## Introduction

Bipolar disorder (BD) is a disabling illness with a significant economic burden, to the individual and to society (Fagiolini et al. 2013; Ferrari et al. 2016). Depressive, (hypo)manic and mixed phases occur repeatedly with euthymic states in between (Green et al. 2011; Rowland et al. 2013; Dodd et al. 2019; Panchal et al. 2019). Affected patients suffer from impaired cognitive emotional processing and maladaptive regulation of emotions (Duggirala et al. 2020). Further, they show significant cognitive impairments in other important areas, like executive function, memory, working memory, and attention (Robinson et al. 2006; Bortolato et al. 2015; Kurtz and Gerraty 2009; King et al. 2019). A substantial proportion of patients continues to experience emotional and cognitive impairments in the euthymic interval (Vöhringer et al. 2013; Cullen et al. 2016; Judd et al. 2008; Mann-Wrobel et al. 2011). Yet, it is still unclear to what extent euthymic patients differ from acutely ill patients in emotional cognition and in strategies of emotion regulation. Distorted or maladaptive cognition is key to the development and maintenance of mood disorders (Grabowski et al. 2019). Therefore, understanding the specific nature of cognitive processing of emotional stimuli and its relationship to emotion regulation abnormalities in BD may be helpful to improve prevention, diagnostics, and treatment.

Altered attention to valanced stimuli, i.e., attentional bias, might be one mechanism that contributes to the emotional dysfunction in bipolar patients (Mathews and MacLeod 2005). For unipolar depression, there is clear evidence that acutely depressed patients focus their attention more on emotionally negative stimuli and away from positive information, as proved, for example, with the dot-probe task (DPT) (Leppänen 2006; Winer and Salem 2016). This negativity bias can persist even after remission of depression (Joormann and Gotlib 2007). For BD, attentional biases are less researched. For acutely depressed bipolar patients, some studies showed an attentional bias towards negative stimuli (Lyon et al. 1999; García-Blanco et al. 2013) whereas other authors report a bias towards emotionally relevant stimuli, regardless of negative or positive valence (Leyman et al. 2009) or a general bias away from emotional stimuli (Jongen et al. 2007). In clinically stable bipolar patients, there is evidence that a bias towards negatively valanced information is also present during the euthymic phase (Gopin et al. 2011)—reflecting a trait effect. Studies found no difference at all for emotional information processing between (euthymic) bipolar patients and healthy controls (Peckham et al. 2016a; Rubinsztein et al. 2006).

Emotion regulation, or coping strategies, are important to control the intensity, duration, experience, and expression of emotions (Gross 1998). Inadequate emotion regulation increases the risk for developing mental problems. Previous studies on emotion regulation in a bipolar sample with on average mild depressive symptoms showed that patients responded more frequently to negative events by catastrophizing, self-blame, and rumination, and that they made less use of putting into perspective than healthy participants (Green et al. 2011; Rowland et al. 2013). Investigations in euthymic patients suggest that even in remission, patients tend to use maladaptive emotion regulation strategies in response to negative experiences (Wolkenstein et al. 2014; Thomas et al. 2007). One study in manic patients showed that they made more use of active-coping and risk-taking than remitted patients, depressed patients, or healthy controls (Thomas et al. 2007).

It could be shown that bipolar patients with manic episodes in their medical history tend to amplify positive emotions (e.g., by thinking about how happy and strong they feel) in response to positive affect compared to individuals without prior manic episodes (Johnson et al. 2008). Thus, it is probable that the predominant polarity and maybe also the number of previous episodes may have an influence on emotion regulation and attentional biases. Predominant polarity is a concept introduced in the last decade in bipolar disorder research and assumes that a major part of patients suffers from either predominantly manic or predominantly depressive course. The predominant polarity is operationalized by twice as many episodes of one disease pole than of the other, which applies for at least half of the bipolar patients (Carvalho et al. 2014; Colom et al. 2006). Besides its association with specific clinical characteristics (Carvalho et al. 2014; Colom et al. 2006; Popovic et al. 2012) the predominant polarity of a patient is a predictor for the occurrence of future phases and may have clinical relevance for the choice of mood stabilizing medication (Carvalho et al. 2014; Popovic et al. 2012). The influence of the predominant polarity on emotional regulation and attentional biases has hardly been investigated. Another important aspect which is widely unknown, is the relationship between attentional biases and emotional regulation in BD. There is first evidence that euthymic bipolar patients, who tend to attenuate positive emotions, direct their attention away from positive stimuli, what, however could not be replicated in a subsequent study (Peckham et al. 2016a, b). A better understanding of the relationship between predominant polarity, attentional bias and emotion regulation could be relevant for improving the treatment strategy in the euthymic phase of BD.

In summary, literature indicates that both, distorted attention processes due to stimulus valence and dysfunctional emotion regulation strategies, are present in BD. However, the findings are partly inconsistent, and especially the extent to which the abnormalities persist in the euthymic phase is unclear. Moreover, the connection between attentional biases and dysfunctional emotion regulation as well as the possible influence of predominant polarity are hardly understood. With this study, we pursued the goal to contribute to the clarification of these questions. We recruited bipolar patients in acute depression and in remission and stratified them depending on their predominant polarity. Analogous to the study by Leyman and colleagues (Leyman et al. 2009), we focused on bipolar patients in the acute depressive phase, instead of manic phase, because depressive episodes generally dominate the course of a BD (Judd et al. 2002) and a higher number of previous depressive episodes correlates with greater cognitive deficits (Bearden et al. 2001). For examining selective attention to emotional stimuli, we used the DPT (MacLeod et al. 1986) which has proven to be sensitive to attentional biases in BD (Leyman et al. 2009; Jongen et al. 2007; Jabben et al. 2012). To compare the implicit results of the DPT with explicit perception of the emotional faces, we assessed arousal and valence ratings of the facial expressions. Previous studies on attentional bias in bipolar patients or unipolar depressed patients used different presentation durations of the emotional stimuli (Leyman et al. 2009; Jongen et al. 2007; Jabben et al. 2012; Bradley et al. 1997; Gotlib et al. 2004). Therefore, in the present study, the emotional faces were presented for a short and a longer duration in the DPT.

For acutely depressed patients (BP-acute-D) we assumed a bias towards negative and away from positive stimuli compared to the healthy controls (HC). Since Popovic and colleagues showed that the predominant polarity is a predictor for the occurrence of future phases (Popovic et al. 2012), we assumed that residual symptoms according to the predominant polarity remain in the euthymic interval which could cause a mood-congruent attentional bias. Therefore, we expected a bias towards positive and away from negative stimuli for euthymic patients with a predominant manic polarity (BP-euth-M) and a bias towards negative and away from positive stimuli for euthymic patients with a predominant depressive polarity (BP-euth-D). Furthermore, we hypothesized that the BP-acute-D will show slower reaction times than the HC since psychomotor retardation is a common finding in depression (Bennabi et al. 2013). Regarding emotion regulation, we assumed that the patient groups and especially the BP-acute-D will score higher in catastrophizing, self-blame, and rumination, and lower in putting into perspective, than the HC.

## Methods

### Recruitment and sample description

The study was conducted in the Department for Psychiatry, Psychosomatics and Psychotherapy of the University Hospital Würzburg. The euthymic patients with manic or depressive predominant polarity were recruited by means of the patient register of the BD program of the Department for Psychiatry, Psychosomatics and Psychotherapy. A total of 72 participants (aged 21 to 68 years, 35 females) were recruited, of which 20 were healthy controls and 52 were clinically ascertained and diagnosed patients with a bipolar affective disorder I or II according to the criteria of the ICD-10. Participant groups were matched for sex. The groups did not differ in terms of age, education (number of school years), and handedness (see Table 1).

**Table 1:** Demographic variables of the four groups

	BP-acute-D (*N* = 20)	BP-euth-D (*N* = 19)	BP-euth-M (*N* = 13)	HC (*N* = 20)	χ2/ *F*	*p*	Post-hoc tests
Sex (*n*)					.13	.988	–
Female	10	9	7	10			
Male	10	10	6	10			
Handedness (*n*)					2.72	1	–
Right	18	17	12	18			
Left	2	1	1	1			
Relearned left	0	1	0	1			
Age (*M, SD*)	43.9 (15.8)	47.4 (13.4)	46.5 (10.2)	44.0 (15.1)	.31	.822	–
School years (*Mdn, IQR*)	10.0 (3.5)	13.0 (3.0)	10.0 (2.5)	12.5 (3.0)	4.08	.253	–
IQ^a^ verbal (*Mdn*, *IQR*)	100.0 (10.0)	104.0 (24.0)	104.0 (20.0)	112.0 (16.0)	9.54	.023*	BP-acute-D < HC (z = 2.93, p = 0.02)^b^

*BP-acute-D* patients in an acute depressive phase, *BP-euth-D* euthymic patients with a predominant depressive polarity, *BP-euth-M* euthymic patients with a predominant manic polarity, *HC* healthy controls

^a^Multiple-choice-vocabulary-test-B (Mehrfachwahl-Wortschatz-Intelligenztest; MWT-B)

^b^Dunn-Bonferroni-Tests with adjustment for multiple comparisons

*p<0.05

Among the bipolar patients, 20 patients were in an acute depressive phase (BP-acute-D) and 32 patients were currently in remission, i.e., euthymic. Before including an acutely depressed patient, the diagnostic criteria for a current depressive episode were evaluated by two independent psychiatrists. In the group of euthymic patients, 19 patients showed a predominant depressive polarity (BP-euth-D), and 13 patients showed a predominant manic polarity (BP-euth-M). All participants were informed about the study and gave written informed consent. The study was in accordance with the Code of Ethics of the World Medical Association (Declaration of Helsinki) and was approved by the local Ethics Committee (University of Würzburg).

Exclusion criteria were neurological illnesses or brain trauma, as well as substance abuse or dependence. Inclusion criterion for all groups was age 18 to 65 and an IQ > 70. For HC, in addition, mental health was a prerequisite. Inclusion criterion for the patients was the diagnosis of a bipolar-affective disorder, subtype I or II, according to the diagnostic criteria of the ICD-10. Patients who were currently in a mixed state or were diagnosed with a different bipolar subtype (cyclothymia or not otherwise specified), as well as patients with schizoaffective disorders, were not included in the study. The euthymic patients had to be in remission for at least three months (minor episodes within the last three months without stationary treatment were allowed) and had to score below the cut-off values for depression (≤ 12 in the Montgomery Asberg Depression Scale; MADRS) and mania (≤ 5 in the Young Mania Rating Scale; YMRS). For an estimation of intelligence, all participants were tested with the multiple-choice-vocabulary-test-B (Mehrfachwahl-Wortschatz-Intelligenztest; MWT-B) (Lehrl et al. 1995). The BP-acute-D group showed a significantly lower IQ score than the HC group (see Table 1). This is likely to be attributable to an interaction of a selection bias of the HC group (above-average IQ) and IQ deficits in the BP-acute-D group due to depressive symptomatology. The German version of the Mini International Neuropsychiatric Interview (M.I.N.I.) (Ackenheil et al. 1999) was conducted with the control participants to screen for mental disorders.

Personal data were recorded in interviews, and clinical data were collected in an interview with the patients and their relatives (Tables 2 and 3); also, chart reviews were undertaken for all patients. For the BP-acute-D, the duration of the current stationary treatment was assessed. For the euthymic patients, the number of weeks since the beginning of remission, the number and polarity of previous episodes, as well as the polarity of the last experienced episode was assessed (Table 2). Based on the number of previous episodes, the predominant polarity of the patients, i.e., depressive, or manic, was determined. It was defined by twice as many episodes of one disease pole than of the other.

**Table 2:** Number and polarity of episodes to date (euthymic patients)

	BP-euth-D (N = 19)	BP-euth-M (N = 13)	U/χ^2^	*P*
Total number of episodes (*Mdn, IQR*)	7.0 (4.0)	7.0 (8.0)	110.0	.603
Number of depressive episodes (*Mdn, IQR*)	5.0 (3.0)	2.0 (2.0)	14.5	.000***
Number of manic episodes (*Mdn, IQR*)	1.0 (1.0)	5.0 (6.0)	40.5	.001**
Number of mixed episodes (*Mdn, IQR*)	0.0 (1.25)	0.0 (0.5)	101.5	.438
Number of weeks in remission (*Mdn, IQR*) ^a^	104 (206)	156 (240)	101.5	.711
Polarity of the last experienced episode (*n*)^b^			7.25	.013*
Depressive	16 (84%)	3 (33%)		
Manic	3 (16%)	6 (67%)		

*BP-euth-D* euthymic patients with a predominant depressive polarity, *BP-euth-M* euthymic patients with a predominant manic polarity

^a^For two patients of the BP-euth-D group the information about the number of weeks in remission was not available

^b^One patient of the BP-euth-D and four patients of the BP-euth-M had rapid cycling or a mixed episode as last experienced episode

*p<0.05, ***p<0.001

**Table 3:** Clinical variables of the bipolar patient groups

	BP-acute-D (N = 20)	BP-euth-D (N = 19)	BP-euth-M (N = 13)	χ2	*p*	Post-hoc χ^2^ tests with Bonferroni-correction
Bipolar Type^a^				5.87	.053	–
Type I	8 (44%)	9 (47%)	11 (85%)			
Type II	10 (56%)	10 (53%)	2 (15%)			
Age of onset of disease^b^	28.2 (12.5)	27.8 (11.0)	29.1 (10.8)	.16	.926	–
Number of inpatient stays^c^	4.6 (6.2)	3.1 (2.3)	3.5 (2.9)	.99	.611	–
*Medication*^*d*^						
Antidepressants	19 (95%)	12 (63%)	6 (46%)	10.1	.006**	BP-acute-D > BP-euth-M (χ2 = 10.236, p = .009)
Lithium	11 (55%)	12 (63%)	9 (69%)	.71	.702	–
Valproate	5 (25%)	4 (21%)	3 (23%)	.20	1	–
Pregabalin	2 (10%)	1 (5%)	1 (8%)	.57	1	–
Lamotrigine	3 (15%)	1 (5%)	1 (8%)	1.10	.837	–
Antipsychotics	18 (90%)	7 (37%)	10 (77%)	13.2	.001**	BP-acute-D > BP-euth-D (χ2 = 11.965**,** p = .003)
Benzodiazepines	2 (10%)	3 (16%)	-	.29	.661	

*BP-acute-D* patients in an acute depressive phase, *BP-euth-D* euthymic patients with a predominant depressive polarity, *BP-euth-M* euthymic patients with a predominant manic polarity

^a^Number and percentage of patients (for two patients of the BP-acute-D group the information about the bipolar type was not available)

^b^Mean age and SD (for one patient of the BP-acute-D group the information about the age of onset of disease was not available)

^c^N and SD

^d^Number and percentage of patients

**p<0.01

Due to their predominant polarity the two euthymic groups differed in the number of previous depressive and manic phases as well as in the polarity of the last experienced episode. There was no significant difference in the number of weeks since the last episode (depressive or manic) between the BP-euth-D and BP-euth-M (Table 2) or in the period of time since the last depressive (*Mdn* = 142, *IQR* = 224) and manic episode (*Mdn* = 32 weeks, *IQR*: 204) of all euthymic patients regardless of their predominant polarity (U = 38.50, z = − 1.486, p = 0.144).

BP-acute-D had been hospitalized on average for 2.3 (SD 1.8) weeks. In the BP-acute-D group, the proportion of patients taking antidepressants was higher than in the BP-euth-M group (Table 3). The proportion of patients taking antipsychotics (which included quetiapine treatment) was higher in the BP-acute-D group than in the BP-euth-D group (Table 3).

### Data assessment

#### Current affect and emotion regulation

Current affect was assessed in all participants using the German Version of the Positive and Negative Affect Schedule (PANAS) (Watson et al. 1988; Krohne et al. 1996). PANAS is a self-report questionnaire with the dimensions positive and negative affect, consisting of 10 items each. Depressive and manic symptoms of the bipolar patients were assessed with the German version of the MADRS (Montgomery and Asberg 1979; Schmidtke et al. 1985) and the YMRS (Young et al. 1978; Muehlbacher et al. 2011). Emotion regulation strategies were retrieved with the German Version of the Emotion Regulation Questionnaire (ERQ) (Gross and John 2003; Abler and Kessler 2009) and a short version of the German Version of the Cognitive Emotion Regulation Questionnaire (CERQshort) (Garnefski and Kraaij 2006; Loch et al. 2011). The ERQ collects data on emotional experience and expression of feelings, testing two common strategies of emotion regulation: (cognitive) reappraisal and the suppression of emotions. In the general population, a high score in the dimension ‘reappraisal’ was shown to correlate negatively with anxiety and depression, whereas a high score in the dimension ‘suppression’ was shown to correlate positively with anxiety and depression (Wiltink et al. 2011). The CERQshort consists of 18 items and samples data on emotion regulation strategies when dealing with negative experiences. Nine different dimensions are covered: ‘self-blame’, ‘acceptance’, ‘rumination’, ‘positive refocusing’, ‘planning’, ‘positive reappraisal’, ‘putting into perspective’, ‘catastrophizing’, and ‘other-blame’. For the longer version of the questionnaire, the CERQ, it was shown that in the general population, high values in the variables ‘self-blame’, ‘rumination’, and ‘catastrophizing’ correlate positively and high levels in ‘reappraisal’ correlate negatively with symptoms of depression and anxiety disorder (Garnefski and Kraaij 2007).

#### Dot-probe task with emotional faces and stimulus rating

The DPT was performed using the software Presentation® (Neurobehavioral Systems, Inc., Berkeley, CA). The task was presented on a 17-inch monitor and participants were sitting at a distance of approx. 50 cm to the screen. Each trial started with the presentation of a fixation cross for a duration of 500 ms, where participants were asked to focus on, in order to direct the view to the center of the screen. Then two faces, one on the left and one on the right side of the screen, appeared for 250 ms or 1250 ms: an emotional face (either ‘sad’ or ‘happy’) paired with a neutral face. Subsequently, a black dot appeared in place of one of the two faces. In emotion-congruent trials, the dot appeared in place of the emotional face and in emotion-incongruent trials the dot appeared in place of the neutral face. Participants had to indicate the localization of the dot by pressing the key ‘L’ for the right side and the key ‘S’ for the left side on a computer keyboard. The pictures used as stimuli were taken from the Radboud Faces Database, which is freely accessible (Langner et al. 2010). A happy, a sad and a neutral face of 14 female and 14 male actors, each, were selected. Consequently, a total of 84 pictures and 56 different pairings of neutral and emotional pictures were available for the task. To achieve a sufficiently high number of trials, the pairings were repeated once at random. Two runs with the same 112 trials were conducted. In one run the faces were presented for 250 ms and in the other run for 1250 ms. Half of the participants of each group started with the short-stimulus run and the other half with the long-stimulus run. Before the first run, all participants completed a test run of about one minute, which was not included in the data analysis. After the first run, there was a 5-min break.

Following the DPT, all happy and sad faces were once again presented. In a valence rating, participants were asked how pleasant or unpleasant they found the picture (1 = very unpleasant; 9 = very pleasant). In an arousal rating, they were asked to indicate the degree of arousal at the sight of the happy or sad face (1 = not arousing at all; 9 = very arousing).

#### Testing procedure

First, the M.I.N.I. (in the control subjects) and the (clinical) interviews were done. Second, the affective symptoms of the bipolar patients (MADRS, YMRS) were assessed. Then, participants completed the MWT-B, ERQ, CERQshort and the PANAS, in that order. Subsequently, the DPT was performed. The complete testing took about one hour per participant. For data protection reasons, all questionnaires were anonymized. At the end of the test, the control participants and the participating outpatients received an expense allowance of 10 and 15 euros, respectively.

### Data analysis

#### Data preparation and comparisons

Sum scores of YMRS and MADRS were compared between the patient groups. Sum scores of the dimension negative and positive affect of the PANAS, as well as of the dimensions of the ERQ and the CERQshort were compared between all groups. Error trials in the DPT, i.e., trials in which participants did not detect the location of the dot correctly, and trials with reaction times less than 200 ms and greater than 750 ms, were excluded from the analysis. The number of error trials and the number of trials outside the response window were compared between all groups. Individual mean reaction times were calculated for emotion-congruent and emotion-incongruent trials for the pairings neutral-happy and neutral-sad for the two presentation durations. To be able to make a statement about an attention bias towards or away from the emotional stimulus, for each individual the so-called bias score was determined for both emotions and both presentation durations: bias score = reaction time of emotion-incongruent trials minus reaction time of emotion-congruent trials. A positive bias score indicates a distortion of attention towards the emotional stimulus and a negative bias score indicates a distortion of attention away from the emotional stimulus. Reaction times of the emotion-congruency combinations, i.e., happy-congruent, happy-incongruent, sad-congruent, and sad-incongruent as well as the attentional bias scores for happy and sad, were compared between all groups for the two presentation durations separately. Furthermore, in an exploratory approach, the attentional bias scores of the euthymic patients were compared depending on the last experienced phase (manic or depressive). Values of the valence and arousal ratings were compared between all groups, for the sad and happy faces separately.

#### Statistical tests

The data evaluation was carried out with the statistics program SPSS for Windows, 24.0 (SPSS Inc., Chicago, Illinois, USA). The significance level for all statistical tests was p < 0.05 (trend: p < 0.1). Nominal scaled data was compared with Chi-squared Test. All metric-scaled variables were tested for normal distribution using the Shapiro–Wilk test and for homogeneity of variance using the Levene’s test. If one dataset of the to be compared datasets was nonparametric, a nonparametric test was chosen. For comparisons of non-normally distributed data between all participant groups, the Kruskal–Wallis test and post-hoc Dunn-Bonferroni tests with adjustment for multiple comparisons were applied and for post-hoc comparison of trend significance, the Mann–Whitney-U test was applied (Bonferroni corrected for the number of group comparisons). For comparison of normally distributed data between more than two groups, one-way ANOVAs and post-hoc Tukey HSD tests were used. For normally, but not variance-homogenous data, the Welch’s-test with Games-Howell post-hoc tests was applied. The relationship between the bias scores and the scores of the dimensions of the emotion regulation questionnaires was tested for each group separately, using one-tailed bivariate correlations. If both variables were normally distributed Pearson’s correlation coefficient was calculated. If this was not the case Spearman's rank correlation coefficient was calculated. The p-value was Bonferroni adjusted for multiple comparisons (for the number of dimensions of the emotion regulation questionnaires and the number of participant groups).

## Results

### Mood ratings and PANAS

Euthymic patients showed on average low MADRS scores (see Table 4). The scores did not differ for BP-euth-M and BP-euth-D (z = − 0.405, p = 1). BP-acute-D showed scores which were on average in the range of moderate depression severity and significantly higher than in the euthymic patients (BP-acute-D/BP-euth-M: z = − 4.584, p < 0.001; BP-acute-D/BP-euth-D: z = − 5.553, p < 0.001). All three patient groups scored low in the YMRS and there was no significant difference between the groups (see Table 4). In the PANAS, as expected, there were significant group differences (see Table 4). The BP-acute-D reported lower positive affect than the HC (p < 0.001) and the euthymic patient groups (BP-euth-M: p = 0.005; BP-euth-D: p < 0.001). The BP-acute-D showed significantly higher negative affect scores than the HC (z = − 5.227, p < 0.001). Interestingly, the BP-euth-D also showed significantly higher negative affect than the HC (z = − 3.002, p = 0.016).

**Table 4:** Means (SD)/ medians (IQR) for the mood ratings and the current affect

	BP-acute-D (N = 20)	BP-euth-D (N = 19)	BP-euth-M (N = 13)	HC (N = 20)	F /χ2	*p*	Post-hoc Tests
Mood ratings							
MADRS (*Mdn, IQR*)	26.5 (8.5)	3.0 (4.0)	4.0 (8.0)	–	36.6	.000***	BP-acute-D > BP-euth-D & BP-euth-M
YMRS (*Mdn, IQR*)	1.0 (2.75)	1.0 (3.0)	1.0 (2.0)	–	.39	.824	–
Current affect (PANAS)							
PA (*M*, *SD*)	20.6 (5.6)	28.9 (7.0)	27.8 (5.7)	30.2 (5.2)	10.5	.000***	BP-acute-D < BP-euth-D, BP-euth-M & HC
NA (*Mdn, IQR*)	19.0 (8.0)	14.0 (5.0)	13.0 (17.0)	10.0 (1.0)	27.6	.000***	BP-acute-D & BP-euth-D > HC

*BP-acute-D* patients in an acute depressive phase, *BP-euth-D* euthymic patients with a predominant depressive polarity, *BP-euth-M* euthymic patients with a predominant manic polarity, *HC* healthy controls, *MADRS* Montgomery-Asberg Depression Rating Scale, *YMRS* Young Mania Rating Scale, *PANAS* Positive and Negative Affect Scale, *PA* Positive affect, *NA* Negative affect

***p<0.001

### Strategies for emotion regulation

The analysis of the ERQ scores over the groups revealed a difference in both dimensions—‘reappraisal’ (χ^2^(3) = 14.585, p = 0.002) and ‘suppression’ (F(3, 68) = 2.82, p = 0.046). These differences were based on the BP-acute-D showing significantly lower scores in the dimension ‘reappraisal’ (*Mdn* = 23.0, *IQR* = 8.5, z = 3.744, p = 0.001) and significantly higher scores in the dimension ‘suppression’ (*M* = 17.4, *SD* = 4.7; p = 0.049) than the HC (*Mdn* = 28.5, *IQR* = 6.75; *M* = 12.9, SD = 5.3).

Scores of the CERQshort were significantly different between the groups in the dimensions ‘rumination’, ‘positive reappraisal’, ‘putting into perspective’ and ‘catastrophizing’ (see Table 5). Post-hoc tests revealed that in the dimension ‘rumination’, the BP-euth-M and the BP-acute-D showed higher scores than the HC, in the dimension ‘positive reappraisal’, the BP-acute-D showed lower values than the HC, in the dimension ‘putting into perspective’, the BP-acute-D as well as the BP-euth-D scored lower than the HC and in the dimension ‘catastrophizing’, the BP-acute-D as well as the BP-euth-D showed higher values than the HC. Post-hoc tests for the trend significances revealed that in the dimension ‘self-blame’ BP-euth-M showed higher scores than the HC, in the dimension ‘positive refocusing’, BP-acute-D scored lower than the HC and BP-euth-M and in the dimension ‘planning’ BP-acute-D showed lower scores then the HC.

**Table 5:** Mean scores (SD)/ median scores (IQR) for the dimensions of the CERQshort

	BP-acute-D (N = 20)	BP-euth-D (N = 19)	BP-euth-M (N = 13)	HC (N = 20)	F /χ2	*p*	Post-hoc Tests
Rumination (*Mdn, IQR*)	7.0 (2.8)	6.0 (4.0)	7.0 (2.0)	4.0 (1.0)	χ2 (3) = 15.2	.002**	BP-euth-M (z = − 2.801, p = .031) & BP-acute-D (z = − 3.662, p = .002) > HC
Positive reappraisal (*Mdn, IQR*)	4.0 (2.5)	6.0 (3.0)	6.0 (2.5)	7.0 (3.0)	χ2 (3) = 10.0	.019*	BP-acute-D (z = 2.824, p = .028) < HC
Putting into perspective (*M, SD*)	5.8 (1.7)	5.5 (1.5)	5.8 (1.6)	7.2 (1.5)	F (3,68) = 4.67	.005**	BP-euth-D (p = .006) & BP-acute-D (p = .032) < HC
Catastrophizing (*Mdn, IQR*)	5.0 (3.0)	4.0 (3.0)	4.0 (3.0)	3.0 (2.0)	χ2 (3) = 15.3	.002**	BP-euth-D (z = − 2.937, p = .02) & BP-acute-D (z = − 3.577, p = .002) > HC
Self-blame (*M, SD*)	5.1 (2.1)	5.1 (2.0)	6.2 (2.24)	4.3 (1.7)	F (3,68) = 2.49	.068	BP-euth-M (p = .039) > HC
Positive refocusing, (*Mdn, IQR*)	4.0 (1.0)	4.0 (2.0)	5.0 (2.0)	5.0 (2.8)	χ2 (3) = 7.78	.051	BP-acute-D < HC (z = − 2.4, p = .018) & BP-euth-M (z = − 2.17, p = .03)
Planning (*Mdn, IQR*)	5.5 (3.8)	6.0 (4.0)	6.0 (2.5)	8.0 (2.5)	χ2 (3) = 7.39	.060	BP-acute-D < HC (z = − 2.54, p = .011)

*BP-acute-D* patients in an acute depressive phase, *BP-euth-D* euthymic patients with a predominant depressive polarity, *BP-euth-M* euthymic patients with a predominant manic polarity, *HC* healthy controls

*p<0.05, **p<0.01

### Dot-probe task

#### Trial exclusion and error rate

The average error rate and the average number of trials outside the response window was 1.4% each. The four groups did not differ in the number of error trials (χ^2^(3) = 3.525, p = 0.317), but in the number of trials outside the response window (χ^2^(3) = 16.641, p = 0.001). Post-hoc tests revealed that the BP-acute-D (z = − 3.682, p = 0.001) as well as the BP-euth-D (z = − 3.138, p = 0.010) responded more often outside the response window than the HC. The total of error trials and trials outside the response window (2.8%) were removed and the data analyses were performed on the remaining 97.2% of trials.

#### Comparison of reaction times and bias scores

**Fig. 1:**
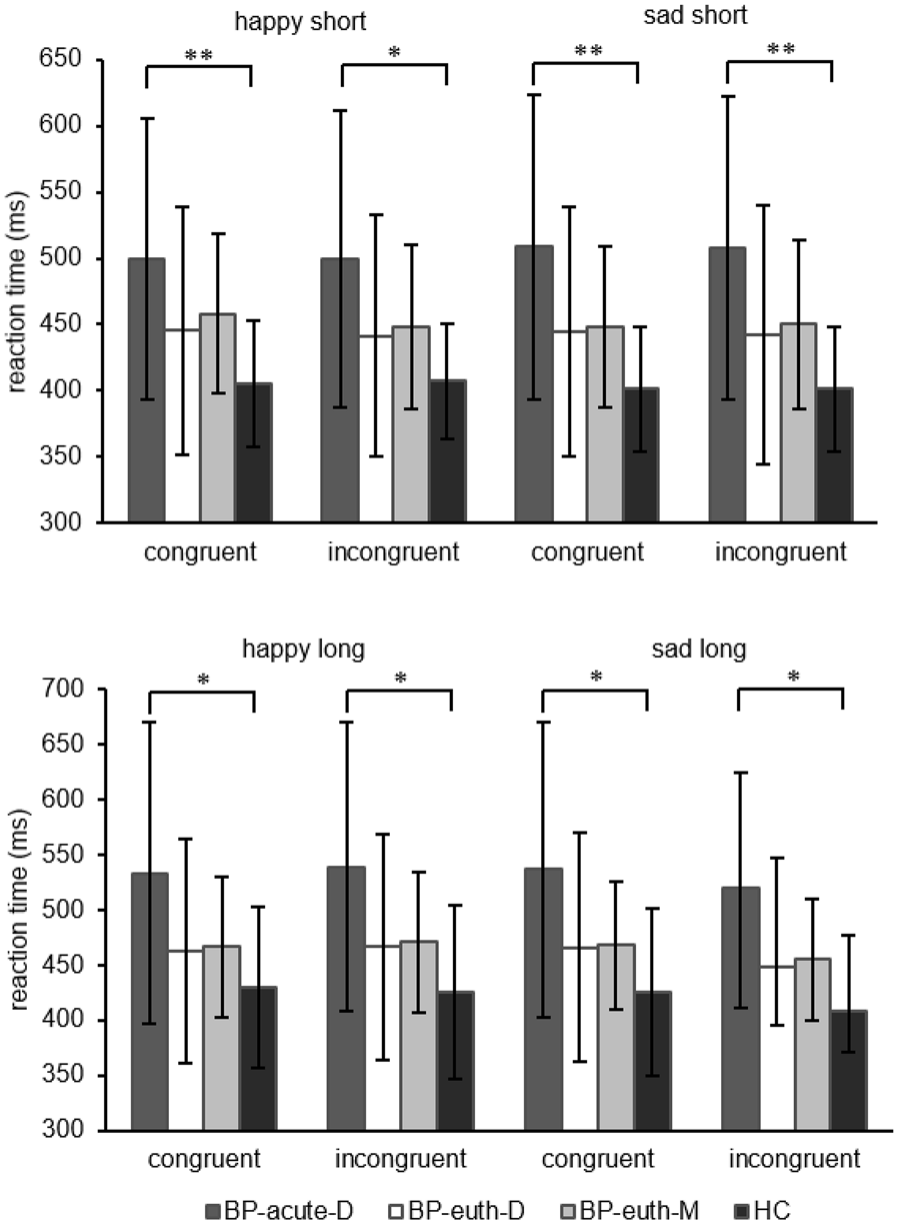
Reaction times in the dot-probe task with a stimulus presentation duration of 250 ms (upper plot) and 1250 ms (lower plot). Plotted are mean values (± SD) except for the condition ‘invalid sad long’ where medians (± quartile) are shown. *BP-acute-D* patients in an acute depressive phase, *BP-euth-D* euthymic patients with a predominant depressive polarity, *BP-euth-M* euthymic patients with a predominant manic polarity, *HC* healthy controls

Reaction times differed for all four emotion-congruency combinations over the four groups for both presentation durations (see Table 6; Fig. 1). The subsequent post-hoc tests showed that the BP-acute-D reacted significantly slower than the HC in all emotion-congruency combinations and for both presentation durations.

**Table 6:** Group comparisons of reaction times

Presentation duration	Condition	Congruency	Welch’s F/χ2	*p*	*p* for BP-acute-D vs. HC
Short	Happy	congruent	5.53	.003**	.006**
		incongruent	4.65	.008**	.011*
	Sad	congruent	5.85	.002**	.004**
		incongruent	5.83	.003**	.004**
Long	Happy	congruent	3.01	.043*	.027*
		incongruent	3.71	.020*	.012*
	Sad	congruent	3.54	.024*	.015*
		incongruent	9.46	.024*	.013*

*BP-acute-D* patients in an acute depressive phase, *HC* healthy controls

*p<0.05, **p<0.01

**Fig. 2:**
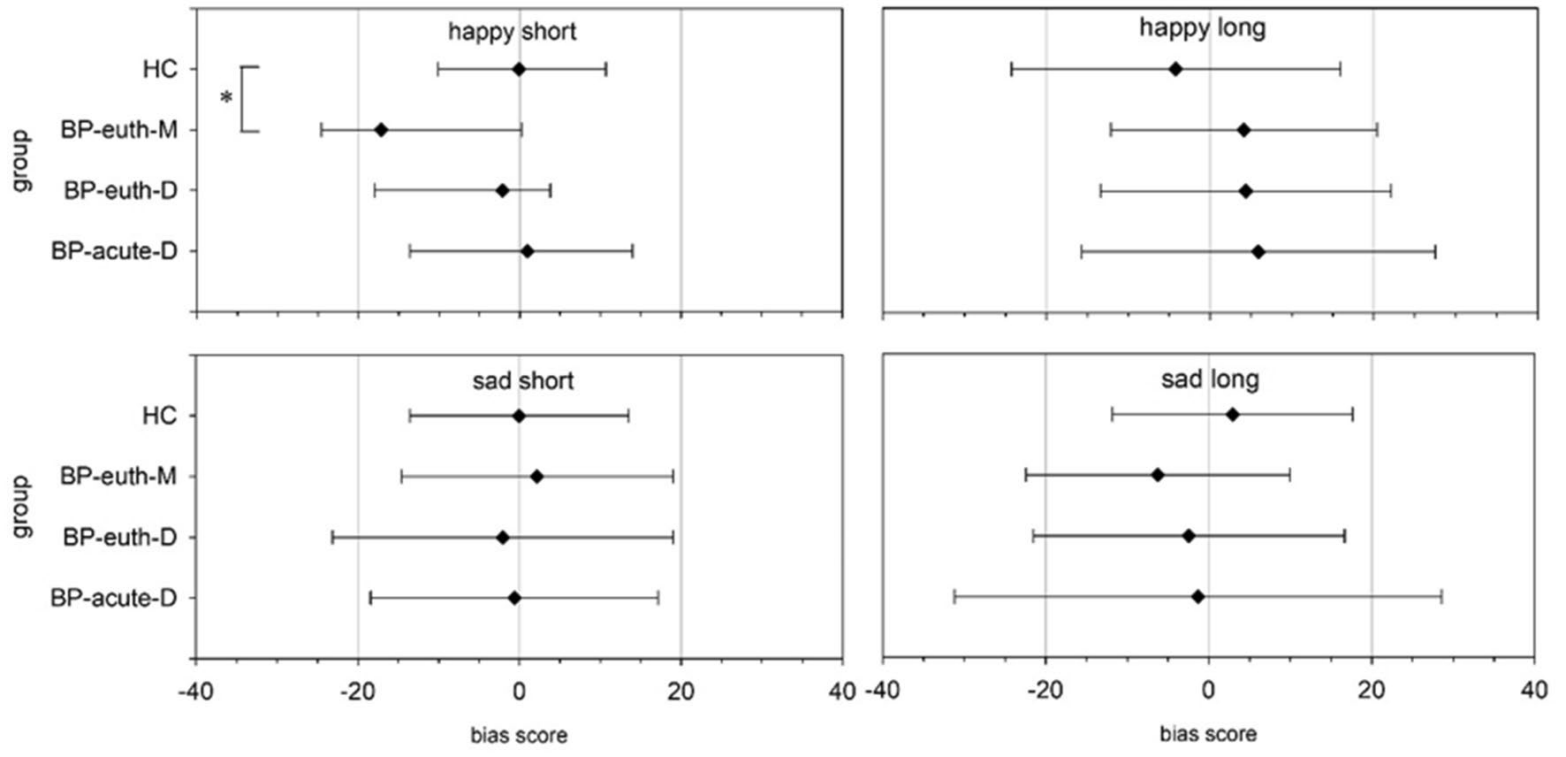
Attentional bias scores in the dot probe task with happy and sad faces with a presentation duration of 250 ms (short) and 1250 ms (long). For the ‘happy short’ condition medians (± quartile) and for the other conditions means (± SD) are plotted. *BP-acute-D* patients in an acute depressive phase, *BP-euth-D* euthymic patients with a predominant depressive polarity, *BP-euth-M* euthymic patients with a predominant manic polarity, *HC* healthy controls

The attentional bias scores showed no significant difference between the groups neither for the short (happy: χ^2^(3) = 7.01, p = 0.072; sad: F(3, 68) = 0.155, p = 0.926), nor the long presentation duration (happy: F(3, 68) = 1.095, p = 0.357; sad: F(3, 68) = 0.515, p = 0.674; see Fig. 2). Since the condition ‘happy’ with short presentation duration showed a trend for significance, we here performed post-hoc group comparisons. The attentional bias scores differed significantly between the BP-euth-M and the HC (U = 55.0, p = 0.03, r = − 0.48), with the BP-euth-M showing a more negative bias score than the HC. For the other group comparisons, there was no significant difference (HC vs. BP-euth-D: U = 145.5, p = 1, r = − 0.2; HC vs. BP-acute-D: U = 199, p = 1, r = − 0.004; BP-euth-M vs. BP-euth-D: U = 88, p = 1, r = − 0.24; BP-acute-D vs. BP-euth-D: U = 157, p = 1, r = − 0.148; BP-euth-M vs. BP-acute-D: U = 82, p = 0.48, r = − 0.161). The attentional bias scores of the euthymic patients in dependence on the last experienced phase (manic or depressive) showed no significant difference for emotion or presentation duration (p’s > 0.05).

### Correlations between emotion regulation and bias scores

**Fig. 3:**
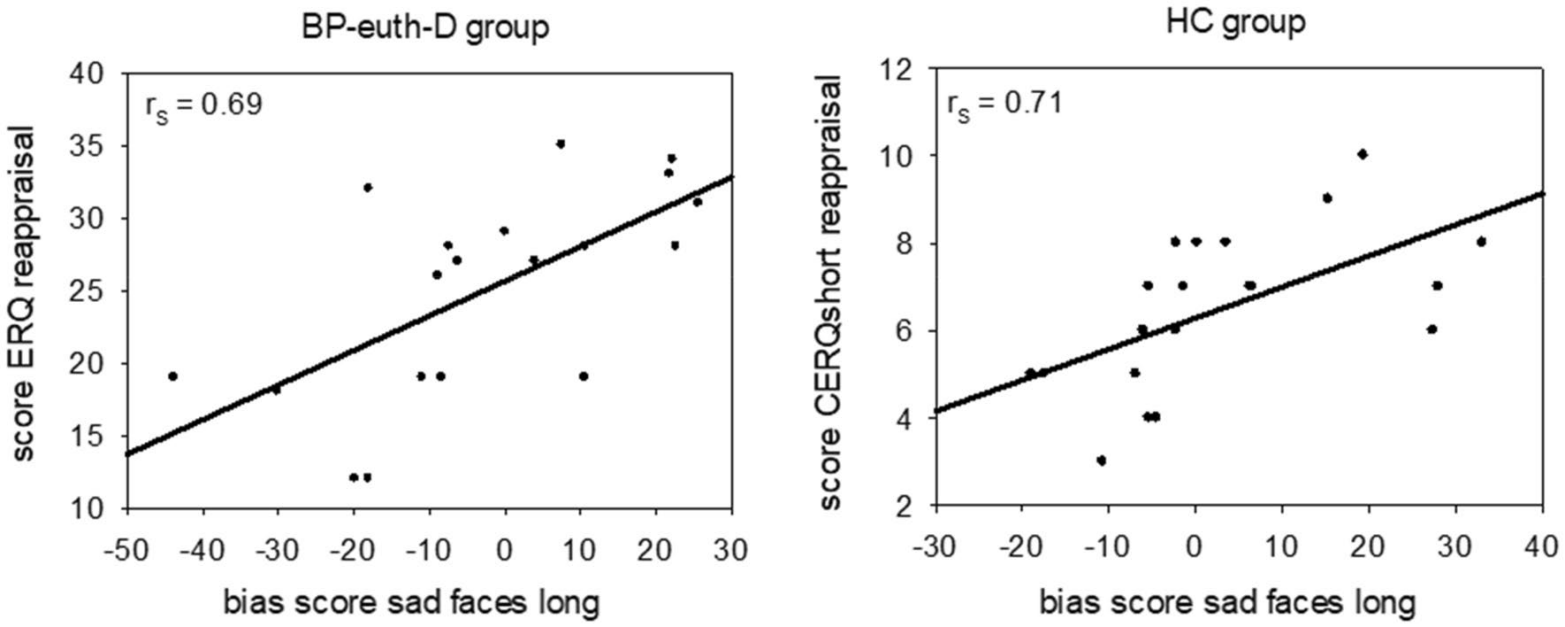
Positive Spearman's rank-order correlations between bias scores for the sad faces with a presentation duration of 1250 ms and the dimension reappraisal (from the ERQ for the BP-euth-D group and from the CERQshort for the HC group). BP-euth-D = euthymic patients with a predominant depressive polarity; HC = healthy controls

Spearman's correlations revealed a strong, positive correlation between the bias score for sad faces, which were presented for 1250 ms, and the dimension reappraisal of the ERQ for the BP-euth-D (r_s_ = 0.687, n = 19, p = 0.025, see Fig. 3). For the HC, there was a strong, positive correlation between the bias score for sad faces, which were presented for 1250 ms, and the dimension reappraisal of the CERQshort (r_s_ = 0.714, n = 20, p = 0.009, see Fig. 3).

### Valence and arousal rating

**Fig. 4:**
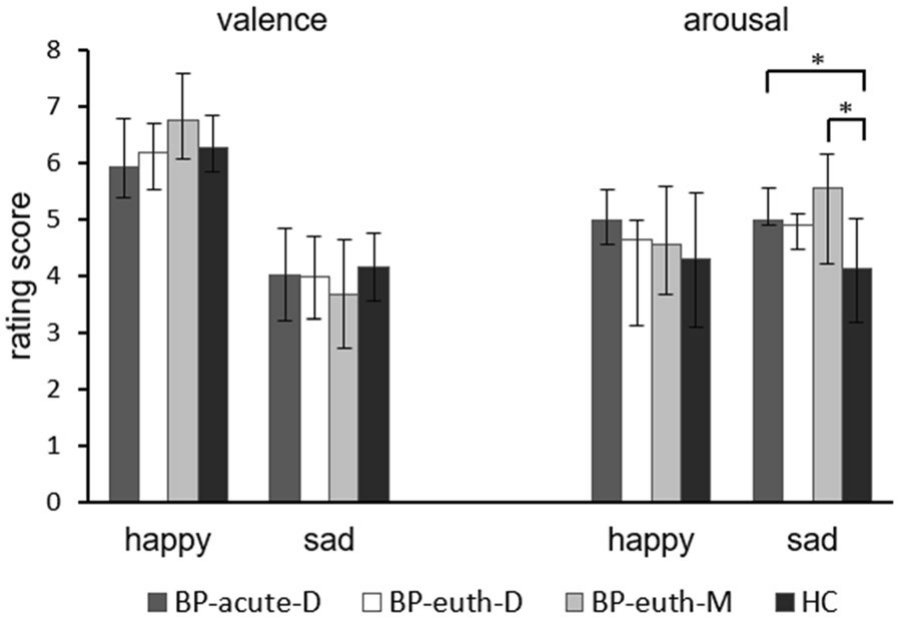
Rating scores of face pictures regarding valence (1 = very unpleasant; 9 = very pleasant) and arousal (1 = not arousing at all; 9 = very arousing). For the ‘valence sad condition’ means (± SD) and for the other conditions medians (± quartile) are plotted. *BP-acute-D* patients in an acute depressive phase, *BP-euth-D* euthymic patients with a predominant depressive polarity, *BP-euth-M* euthymic patients with a predominant manic polarity, *HC* healthy controls

There was a significant group difference in the arousal rating of sad faces (χ^2^(3) = 12.6, p = 0.005, see Fig. 4). Post-hoc tests showed that the BP-acute-D (z = − 2.915, p = 0.021) and the BP-euth-M (z = − 3.032, p = 0.015) showed higher arousal ratings then the HC. Comparisons for arousal ratings of happy faces and for valence over the four groups were not significant (valence sad: F(3, 68) = 1.039, p = 0.381; valence happy: χ^2^(3) = 6.312, p = 0.097; arousal happy: χ^2^(3) = 3.486, p = 0.323).

## Discussion

### Higher negative affect scores in acutely depressed bipolar patients and euthymic patients with depressive predominant polarity

The mood disturbances of acutely depressed bipolar patients are reflected in the negatively distorted PANAS scores. Interestingly, not only acutely ill patients were found to have higher negative affect scores than HCs but also euthymic patients with depressive predominant polarity. Whether this might reflect sub-threshold affective symptoms as a residual effect of acute episodes or as an enduring personality trait remains to be established. Similar findings were reported by Van der Gucht and colleagues who compared PANAS scores in bipolar patients and healthy controls (Gucht et al. 2009). As in our study, the depressed patients reported less positive affect and higher negative affect than the control group. The euthymic patients who were not grouped according to their predominant polarity reported higher negative affect than the control group. Given our results, it can be assumed that this effect was driven by the euthymic patients with predominant depressive polarity. On the other hand, Lomax and colleagues did not report significant differences in current positive or negative affect for euthymic bipolar patients compared to a healthy control group (Lomax et al. 2009).

### Use of maladaptive emotion regulation strategies in the acute depressive and euthymic phase

The present work shows very clearly that bipolar patients, especially in the acute depressive phase, but also in the euthymic phase, tend to use dysfunctional emotion regulation strategies. These results are consistent with numerous previous studies which showed emotion regulation deficiencies in bipolar patients in terms of increased use of maladaptive strategies and less frequent use of functional strategies (Green et al. 2011; Rowland et al. 2013; Dodd et al. 2019; Lima et al. 2018). The more frequent use of maladaptive strategies also persists in the euthymic phase (Wolkenstein et al. 2014; Thomas et al. 2007; Oh et al. 2019). Concerning the suppression of emotions which was reported by the BP-acute-D in our study there is evidence that individuals who suppress their emotions have a higher risk for depressive and anxiety disorders (Dodd et al. 2019; Gross and John 2003; Campbell-Sills et al. 2006). Our study is the first to investigate whether euthymic patients with depressive and manic polarity differ in emotion regulation strategies. The two euthymic groups did not differ significantly in any dimension of emotion regulation. However, their reported dysfunctional behavior applied to different dimensions of emotion regulation what suggests distinct deficits in these two patient groups. Again, it needs to be determined whether this is an effect of the last affective episode, or a trait going along with the disease as such.

### Poorer response capacity in acutely depressed bipolar patients

As expected, BP-acute-D showed slower reaction times than the HC in all conditions. These results are consistent with results from other neuropsychological tests, like the Speed and Capacity of Language Processing Test and the Digit Symbol Substitution Test (Malhi et al. 2007; Gallagher et al. 2014). In our study, reaction times of the euthymic patients differed statistically neither from the BP-acute-D nor from the HC but were numerically longer than for the HC indicating a persistence of minor deficits. Previous studies using neurocognitive tests in euthymic patients provided mixed results (Malhi et al. 2007; Hulvershorn et al. 2012; Wilder-Willis et al. 2001; Fleck et al. 2005). This goes in line with a review by Cullen and colleagues which suggests that the prevalence of impaired speed and reaction time in euthymic individuals is about one third (Cullen et al. 2016). One aspect that must be considered is a potential medication effect that may impact cognitive speed as measured by proxy with reaction times; this might be of relevance especially for acutely depressed patients who took significantly more antidepressants or antipsychotics than the euthymic patients with manic polarity or depressive polarity. Indeed, there is evidence that antidepressant doses correlate negatively with reaction times (Kalb et al. 2006).

### Attentional bias away from happy faces in euthymic patients with manic predominant polarity

Our assumption that acutely depressed patients will show a bias towards negative stimuli could not be confirmed in the present study. An absence of a bias towards sad stimuli in acutely depressed bipolar patients was also reported for an Affective Go/No-go Task by Rubinsztein and colleagues (2006). However, several studies have shown a mood-congruent distortion of attention towards negative stimuli in acutely depressed bipolar patients (Lyon et al. 1999; García-Blanco et al. 2013). Other findings point to an attentional bias away from emotional stimuli (Jongen et al. 2007), or to a bias towards angry as well as happy expressions (Leyman et al. 2009). One possible explanation for these inconsistent results is the usage of different neuropsychological tests and a small sample size in some of the studies. In sum, a clear mood-congruent bias, as seems to be present in unipolar depression (Leppänen 2006; Joormann and Gotlib 2007; Armstrong and Olatunji 2012), cannot be assumed for acute depression in BD.

Based on the hypothesis of Popovic and colleagues that residual symptoms may remain even in the euthymic interval (Popovic et al. 2012) we expected a mood-congruent bias for the euthymic patients, depending on their predominant polarity. However, this assumption proves to be wrong as both BP-euth-M and BP-euth-D patients showed a quite similar pattern in the PANAS: increased negative and reduced positive affect as compared to controls (no significant difference though). Furthermore, our results of the DPT do not support this theory since BP-euth-M directed their attention away from positive stimuli and BP-euth-D showed no attention bias at all. Similar to our finding in the BP-euth-M, some studies in euthymic patients or stable outpatients (not grouped according to predominant polarity) also revealed a negativity bias, i.e., an attentional bias toward negative stimuli or away from positive stimuli (Jongen et al. 2007; Gopin et al. 2011). Interestingly, our finding was only evident for the short presentation duration of 250 ms. This suggests that avoidance of positive stimuli in patients with manic polarity only occurs during early phases of information processing which was also reported in a study by Leyman and colleagues (Leyman et al. 2009). The absence of an effect in the DPT in the BP-euth-D is in accordance with studies about attentional biases which did not find any differences between euthymic patients (not grouped according to predominant polarity) and healthy controls (Peckham et al. 2016a; Jabben et al. 2012). The fact that patients with manic polarity directed their attention away from happy faces in the present study can be interpreted in several ways. It is possible that patients with predominant manic polarity suffer from subdepressed mood and negative cognitive schemes also in the euthymic interval, as it is suggested that manic state is a “defense” or counterreaction to underlying depressive tendencies (Alloy et al. 2006). Alternatively, the bias away from positive stimuli could be interpreted as a protective mechanism against potential triggers of mania. Future studies with a larger sample, also including patients in an acute mania, should be conducted to confirm our finding. Moreover, it should be investigated whether avoidance of positive stimuli protects euthymic patients with manic predominant polarity efficiently against a disease relapse and if this strategy could be integrated in psychotherapeutic interventions.

### Correlation between attentional bias and emotion regulation

In the BP-euth-D and HC, increased reported use of reappraisal in the emotion regulation questionnaires was related to a stronger distortion of attention towards sad faces in the condition of longer face presentation in the DPT. As the paradigms and presentation times of the stimuli in previous studies differ greatly, the results are only comparable to a limited extent. Contradictory to our results, previous studies found a positive relation of the use of reappraisal and the reduction of negative attention bias (Sanchez et al. 2016; Vanderhasselt et al. 2013). However, results by Adam and colleagues go in the same direction as our results (Adam et al. 2014). Studies suggest that using reappraisal for negative input reduces experience of negative affect (Ochsner et al. 2002) and increases distress tolerance (Naragon-Gainey et al. 2017). The increased attentional bias for negative stimuli of participants reporting more use of reappraisal might reflect an engagement due to the application of reappraisal and/or a longer tolerance of the sad stimuli.

### Higher arousal to sad faces in acutely depressed bipolar patients and euthymic patients with manic predominant polarity

The BP-acute-D as well as the BP-euth-M reported significantly higher arousal than the HC when presented with the sad faces used in the DPT. For the BP-acute-D, this goes in line with a study by Branco and colleagues showing that bipolar patients with an average mild depressive symptomatology perceived the intensity of negative facial stimuli stronger than healthy subjects (Branco et al. 2018). This finding is corroborated by a neuroimaging study which revealed an increased neural activity to negative, but also to positive stimuli in this patient group (Lawrence et al. 2004). Contradictory to our results of the BP-euth-M there is evidence for a decreased neural activity to sad faces in mania (Lennox et al. 2004). Our data on the arousal level and the PANAS scores argue that regardless of the predominant polarity depressive traits are more discernible than manic traits in phases of remission. Whether this is specific to our sample remains to be determined but it fits to studies suggesting that disease burden of bipolar disorders is mostly attributable to depression. It is assumed that stimuli which are perceived as particularly arousing are processed preferentially (Mather and Sutherland 2011) indicating that there is a connection between emotional arousal and selective attention (Lee et al. 2014; Phelps 2006). However, this hypothesis could not be confirmed in our work since acutely depressed and euthymic patients with manic polarity showed increased arousal to sad faces, but no attentional bias towards them. Further studies are needed to shed more light on these connections in bipolar disorder.

### Limitations

A strength of the present study is that both, acutely depressed and euthymic bipolar patients, were examined. Particularly, euthymic patients were grouped according to their predominating polarity which enabled a more precise comparison of cognitive deficits in different subtypes of the disorder. Nevertheless, one should consider some limitations when interpreting the results. First, it should be noted that information about the clinical characteristics was collected through interviews with the patients and is therefore subjective. Although the polarity of the last episode has not had a significant effect on the attentional bias score and the period of time since the last depressive and manic episode of all euthymic patients did not differ significantly, a time-related effect of the last episode on the attentional bias cannot be completely ruled out. Furthermore, the whole sample size of the study is rather small especially in the group of BP-euth-M since bipolar patients are more likely to suffer from depressive than manic episodes (Judd et al. 2002). One must consider that the 52 bipolar patients include 22 patients with Bipolar Disorder Type II leading to a less homogenous cohort. Also, the classification of the euthymic patients into their predominant polarity further reduces the sample size of the subgroups and the statistical power. Another limitation is that only patients in acute depression, but not in mania, were included in the study, the reason being that only very few patients are both willing and able to conduct such studies during an acute manic episode. Thus, for getting a more complete understanding of emotion regulation and attentional biases in BD, further studies including a group of manic patients and greater sample sizes with a higher statistical power should be conducted. When interpreting our results, one must consider that especially the acutely depressed patients took several types of psychotropic drugs. Since some studies point to a negative effect of mood-stabilizing drugs on cognitive performance (Bilderbeck et al. 2017; Holmes et al. 2008) an influence of drug therapy cannot be ruled out. Furthermore, it should be noted that the present work exclusively investigated which emotion regulation strategies were applied to negative, but not to positive events and feelings. There is evidence that patients whose thoughts repeatedly revolve around positive experiences are more likely to switch into a manic phase (Gruber et al. 2011) or exhibit hypomanic symptoms (Raes et al. 2010). Therefore, the way euthymic patients deal with both, negative and positive affect, should be considered in future studies (Wolkenstein et al. 2014) to identify specific maladaptive emotion regulation mechanisms that could help to predict depressive or manic phases.

## Conclusions

This study contributes to the understanding of emotion perception and regulation in BD and provides valuable impulses for future research. It is one of few studies where euthymic patients were grouped according to their predominant polarity. Our results demonstrate that patients with predominant depressive and manic polarity show diverse cognitive patterns which highlights the importance of characterizing euthymic patients in more detail. We found that euthymic bipolar patients with predominant manic polarity showed a cognitive bias away from positive stimuli in a DPT and reported more arousal to negative stimuli whereas euthymic bipolar patients with predominant depressive polarity reported more negative affect and more dysfunction in emotion regulation strategies than euthymic bipolar patients with predominant manic polarity. Our negative finding in the DPT for the acutely depressed group indicates that there is no clear evidence for an attentional negativity bias in depressed bipolar patients. The attention away from positive stimuli within the BP-euth-M can be interpreted as a protection mechanism for triggers of mania.

For future studies, we recommend the examination of euthymic patients with particular respect to their predominant polarity in more detail since dysfunctional emotional cognition and regulation can trigger a relapse into a depressive or manic phase (Wolkenstein et al. 2014) and thus further insights could be helpful to prevent relapses (Rheenen et al. 2015). Likewise, longitudinal studies with bipolar patients in the different phases of the disease would contribute to a deeper understanding of dysfunctional emotional cognition and regulation. This would also allow to better determine whether distorted attention processes and dysfunctional coping mechanisms are trait or state characteristics. The identification of such traits could help to decipher genetic markers associated with a higher risk for the development of BD (e.g., polygenic risk scores for mania or depression). Since studies with depressive patients prove that the processing of emotional information can be influenced by cognitive training (MacLeod et al. 2002), it should be examined in more detail how bipolar patients benefit from such therapeutic interventions.

## Data Availability

The datasets used and/or analyzed during the current study are available from the corresponding author on reasonable request.

## Abbreviations

BD: Bipolar disorder
BP-acute-D: Bipolar patients in a depressive state
BP-euth-M: Euthymic patients with manic predominant polarity
BP-euth-D: Euthymic patients with depressive predominant polarity
HC: Healthy control participants
DPT: Dot-probe task
MADRS: Montgomery Asberg Depression Scale
YMRS: Young Mania Rating Scale
MWT-B: Multiple-choice-vocabulary-test-B; Mehrfachwahl-Wortschatz-Intelligenztest
PANAS: Positive and Negative Affect Schedule
ERQ: Emotion Regulation Questionnaire
CERQshort: Short Cognitive Emotion Regulation Questionnaire
